# Manifestation of Health Denialism in Attitudes toward COVID-19 Vaccination: A Qualitative Study

**DOI:** 10.3390/vaccines11121822

**Published:** 2023-12-06

**Authors:** Iwona Młoźniak, Urszula Zwierczyk, Elżbieta Rzepecka, Mateusz Kobryn, Marta Wilk, Mariusz Duplaga

**Affiliations:** 1 Department of Health Promotion and e-Health, Institute of Public Health, Faculty of Health Sciences, Jagiellonian University Medical College, 31-066 Krakow, Poland; iwona.mlozniak@uj.edu.pl (I.M.); urszula.zwierczyk@uj.edu.pl (U.Z.); m.kobryn@doctoral.uj.edu.pl (M.K.); martam.wilk@student.uj.edu.pl (M.W.); 2 Department of Epidemiology and Population Studies, Institute of Public Health, Faculty of Health Sciences, Jagiellonian University Medical College, 31-066 Krakow, Poland; elzbieta.brzezicka@uj.edu.pl

**Keywords:** health denialism, conspiracy theories, pandemic, COVID-19, preventive behaviors, COVID-19 vaccination, SARS-CoV-2, coronavirus

## Abstract

Science denialism is characterized by the refusal to accept existing consensus and available evidence. Typical strategies denialists employ include spreading conspiracies, selective use of information, relying on fake experts, or general fallacies in logic. A flood of misinformation, fake news, and conspiracy theories accompanied the COVID-19 pandemic. Simultaneously, it was a subject of many denialistic opinions, from denying the existence of the epidemic challenge to claims that questioned the safety and effectiveness of the COVID-19 vaccines. This study’s main aim was to assess the manifestations of denialism in attitudes toward the preventive measures recommended during the pandemic, with a special focus on vaccination. In-depth interviews were conducted with fifty representatives of the general population, demonstrating diversified opinions about COVID-19 vaccines and other preventive behaviors. The interviews were performed face to face in participants’ houses or at other places they identified as convenient. Some of the interviewees preferred to do the interview via teleconference. The interviews were carried out from November 2022 to March 2023. The interviewees were recruited initially by convenience, and in further stages, the snowball technique was used. The interviewees were residents of four main administrative districts in Poland. Out of 50 participants, 26 were males, 29 were between 18–40, 16 were inhabitants of rural areas, and 28 had a university level of education. The interviews were based on a semi-structured guide that addressed, in addition to views about the origin of the new coronavirus, respondents’ attitudes toward vaccination and sanitary recommendation, the health status of interviewees, their use of healthcare services, and their health behaviors. The interviews were transcribed and analyzed with MAXQDA Analytics Pro 2022 software (Release 22.7.0). Thematic analysis (TA) was applied to the content generated from the interviews. Based on the uptake of the COVID-19 vaccine, the participants were divided into three groups: unvaccinated, hesitant, and vaccinated (18, 4, and 28 interviewees, respectively). The main themes were established based on the TA of the interviews: attitudes toward COVID-19 vaccination, perception of sources of information, and the origin of the new coronavirus. The first theme decidedly drew the greatest attention of the interviewees. There was also a clear relationship between vaccination status and the presence of denialist thinking among interviewees. Interestingly, the role of experts as a key source of information about the pandemic was underlined by study participants. However, the criteria for being an expert differed. The subject of the origin of a new coronavirus was not interesting to interviewees. The analysis of the adherence to preventive measures revealed an interplay of diversified attitudes and motivations. Individuals presenting denialist views most frequently abstained from COVID-19 vaccination. However, such views were also present among those who hesitated or even among those who had been vaccinated. Furthermore, denialism was only one of the determinants of adherence to preventive measures.

## 1. Introduction

The concept of denialism was introduced by the Hoofnagle brothers, who defined it in 2007 as ‘the employment of rhetorical arguments to give the appearance of legitimate debate where there is none’ [1]. According to them, the tactics employed by denialists include spreading conspiracies, selectively citing sources of information, referencing fake experts, creating impossible expectations, and general fallacies of logic. Denialism is employed by those who have no arguments against a scientific consensus or available evidence. The targets of long-standing denialist tactics include global warming, evolution, the Holocaust, 9/11 conspiracies, and, in the health domain, AIDS, the carcinogenic effect of tobacco smoking, and vaccination. The motivations for denialism may include defending business interests by denying that certain products are harmful, rejecting facts that conflict with one’s ideology, and the desire to distinguish oneself from others or to maintain celebrity status.

Hansson described science denialism as one of the two main forms of pseudoscience, alongside the promotion of pseudo-theories, but according to this author, the latter also requires some degree of science denialism [2]. This view remains to some degree, in line with the initial claims of the Hoofnagles, who believed spreading conspiracies to be a key element of denialism [1].

In a commentary published shortly after the Hoofnagles’ post, Diethelm and McKee argued that public health professionals should be aware of denialism and have the capacity to recognize and counteract it [3]. The COVID-19 pandemic proved the validity of their assertion. The number of denialists who rejected the relationship between the emergence of a new coronavirus and a worldwide pandemic, or the existence of the pandemic at all, rose rapidly [4]. Conspiracy theories about the origin of the new coronavirus, its dissemination, and preventive measures also abounded [5].

Conspiracy theories reject the standard explanation of an event and rather attribute it to some hidden groups or organizations that are determined to carry out secret plots. Such theories are a prominent feature of denialism and can be relatively easily measured in society. Many mechanisms that adopt and spread conspiracy theories have been proposed [6]. They are common in various domains, not only in politics but also in science and health. It also seems that conspiracy beliefs in different domains have a tendency to cluster [7]. Some authors have suggested that conspiracy mentality and conspiracy beliefs may be predictors of unfavorable health behaviors, e.g., not adhering to medical recommendations [8]. Many studies conducted during the COVID-19 pandemic confirmed this suggestion [5]. Conspiracy beliefs were a strong predictor of non-adherence to preventive measures, including COVID-19 vaccination [9,10].

From the very beginning, the COVID-19 pandemic was accompanied by the eruption of misinformation, fake news, and conspiracy theories about the origin of the new coronavirus, possible countermeasures, and the severity of the diseases resulting from infection. As early as February 2020, in one report, the World Health Organization (WHO) used the term ‘infodemic’ to name the flood of misinformation exacerbating the epidemiological challenge [11]. Some authors suggested that it could lead to severe consequences [12,13]. Quickly, the COVID-19 pandemic became an exemplary locus of health denialism [14].

Studies performed in the first year of the pandemic revealed that considerable proportions of the populations believed in COVID-19 conspiracy theories [13,15,16]. The most common manifestations of denialism, frequently taking the form of certain specific conspiracies, included the opinions that actually there was no pandemic, the severity of COVID-19 disease was being exaggerated, and its course was mild, and finally, that the restrictions imposed by governments were more devastating to economies than the disease itself [4]. Depending on preferences and contexts, denialists shifted between various theories and opinions.

From the beginning of the COVID-19 pandemic, many determinants of preventive behaviors have been indicated, particularly attitudes and hesitancy about COVID-19 vaccination. In their scoping review in 2021, Biswas et al. identified a whole array of factors related to COVID-19 vaccine hesitancy [15]. They included, apart from sociodemographic factors, a negative perception of vaccine safety, efficacy, and side effects, economic issues, being an ethnic minority, inconsistent messaging from public health organizations, mistrust of the healthcare system and government, political instability, and doubts about the process of vaccine development. Interestingly, the lack of information about vaccines, widespread misinformation, and anti-vaccination movements were also frequently identified by researchers as determinants of increased hesitancy. Recently, Kadafar et al., in a systematic review of systematic reviews, distinguished several categories of factors reported most frequently as determinants of COVID-19 vaccine hesitancy [16]. These categories encompass contextual factors (sex, age, social inequalities), individual and group factors, and vaccine and disease-specific factors. According to this classification, trust in the health system, public health authorities, and governments, as well as a history of vaccination are among individual and group factors. Vaccine-specific factors include concerns about vaccine safety, perceived vaccine barriers, perceived vaccine effectiveness, and concerns about the vaccine’s rapid development. Finally, the last category of disease-specific determinants should include fear of being infected, perceived severity of COVID-19, and knowledge regarding COVID-19.

It should also be underlined that attitudes toward COVID-19 vaccination in society could be influenced by following reports about vaccines’ effectiveness and side effects. Particularly, the emergence of the Omicron variant of SARS-CoV-2 could lead to certain disillusionment due to a significant drop in the effectiveness of available vaccines against the prevalence of symptomatic COVID-19 disease [17]. The review by Mohammed et al. showed that pooled COVID 19 vaccines’ effectiveness against Omicron infection was 28.1% after three months from the primary vaccination, but it declined to 3.9% at six months [18]. Furthermore, reports about the side effects of COVID-19 vaccines could transpire to broader audiences and incite skepticism about vaccination. We should also be aware that doubts about COVID-19 vaccines’ effectiveness and potential side effects were amplified and propelled by anti-vaccination movements, adding to the general misinformation prevalent during the pandemic [19,20].

Studies performed during the COVID-19 pandemic showed clearly that there is a significant relationship between conspiracy beliefs and adherence to preventive measures and attitudes toward COVID-19 vaccination. Romer and Jamieson reported as early as October 2020 that conspiracy beliefs were barriers to controlling the spread of COVID-19 in the USA [21]. Their study showed that conspiracy beliefs about COVID-19 were prevalent and stable over time and prospectively predicted resistance to preventive actions and vaccination. Similar findings were reported for Poland by Duplaga based on a survey performed in 2020 [9]. Believing conspiracy theories predicted lower adherence to the preventive measures recommended during the initial phase of the pandemic. A study performed in October 2021 showed that conspiracy beliefs were a negative predictor of the uptake of COVID-19 vaccination [10]. The negative association between conspiracy beliefs and compliance with preventive behaviors and vaccination willingness during the COVID-19 pandemic was confirmed in many other studies [5,22].

The main objective of the study was to analyze manifestations of denialism in attitudes toward the preventive measures recommended during the pandemic, with a special focus on COVID-19 vaccination. We analyzed the perceived safety and usefulness of the vaccine as well as the motivations to get vaccinated or not. The analysis presented here was also aimed at explaining participants’ views about the origin of the new coronavirus, their perception of the threat of the COVID-19 pandemic, and how they used the information coming from various sources.

## 2. Materials and Methods

This qualitative study was performed within the framework of a broader research project, which employed mixed methods and aimed to explain the relationship between health literacy and susceptibility to health denialism and misinformation. This paper reports the analysis of the participants’ responses to questions about their perception of the pandemic, attitudes toward COVID-19 vaccination, and adherence to other preventive measures. The data was obtained through in-depth interviews (IDIs) conducted with 50 respondents based on a semi-structured guide. The research material was, after transcription, analyzed with the MAXQDA Analytics Pro 2022 software (Release 22.7.0).

Thematic analysis (TA) was used as the method, allowing for the simultaneous use of assumptions originating both from theory and previous research. TA enabled identifying gaps in the knowledge and understanding of the denialistic approach through inductive coding [19]. Whereas the study aimed to explore the beliefs of the researched population with a kind of ‘fresh look’, it was based on prior knowledge of phenomena related to COVID-19 denialism and conspiracy theories [23].

The interview guide was designed for the study by the research team. It contained a list of open-ended questions that should be raised during the interview. The guide was piloted in several subjects before starting the main study. Questions concerned, inter alia, attitudes toward vaccines, the new coronavirus, views on the origins of SARS-CoV-2, and sources of information grouped according to the topic (for the full list of topics and questions, see Table S1).

### 2.1. Participants and Procedure

#### 2.1.1. Sampling

The IDIs aimed to explore denialist attitudes in the studied population. To avoid misinterpretation of the interviews, participants were recruited according to sociodemographic characteristics (gender, education, place of residence, and vocational status) and availability. We wanted the interviewed group to be diverse, with the possibility of including a group with strong denialist attitudes. The interviews were not repeated.

The first participants were recruited by convenience, and in further stages, the snowball technique was applied. The interviewers were required to approach respondents with whom they did not have a close relationship, e.g., kinship or friendship. The researchers who conducted interviews were encouraged to recruit participants who presented diversified sociodemographic characteristics. Finally, 50 interviews were performed, 24 with women and 26 with men. The age of participants ranged from 19 to 69. Interviewees were from four Polish administrative districts (voivodships): Mazowieckie, Małopolskie, Świętokrzyskie, and Podkarpackie. Details of the study group’s sociodemographic characteristics are provided in Table 1. The interviewees were not asked about any details of medical history. This could be seen as a deficiency, as their COVID-19 status could not be considered; however, the authors tried avoid intrusive questions which could influence the course of interviews.

The total number of refusals to take part in the study was 11. In 3 cases, interviewees with strong denialist attitudes refused to take part in the study because of their reluctance toward public health initiatives and their lack of trust in the academic institution represented by the researchers. However, 8 of the 50 interviewees declared that they did not take the COVID vaccine because of their beliefs and a few more took the vaccine only because of some external coercion.

The sample analysis showed that data saturation was reached despite the unequal share of persons with and without denialist attitudes in the interviewed group.

#### 2.1.2. Setting

The interviews were performed from November 2022 to March 2023. Most of the interviews were conducted face to face in participants’ houses or in other places they identified as safe and comfortable, e.g., a cafeteria. Some were conducted via teleconferencing if the interviewee found it more convenient. All the interviews were recorded and transcribed by the interviewers. In the transcriptions, the way participants spoke (grammatical mistakes, repetitions) was preserved. The quotes presented in the article have been adapted for ease of reading (they were translated into English, corrected grammatically, and word duplicates were removed). Transcripts were not returned to participants for comments. Participants were not asked for feedback on the findings.

#### 2.1.3. Quality Criteria

The study was guided by the Consolidated Criteria for Reporting Qualitative Studies (COREQ) [24]. MD designed the study and supervised its execution. The interviews were conducted by EB, MK, MW, UZ, and IM. The team conducting interviews included two sociologists, a historian/archivist working as a research assistant, a dietitian, and a medical doctor. The courses on qualitative methods were part of the curriculum during the university education received by most of them. IM, with the greatest experience in IDIs, conducted several workshops for the team to achieve a common understanding of the research approach. The research team members were employed in the Department of Health Promotion and e-Health on research posts or were PhD students supervised by MD. One person (EB) was employed in another Institute of Public Health unit. IM and EB performed coding and thematic analysis. Information about how the 32 items of COREQ were addressed is provided in the Supplementary File (Table S2).

### 2.2. Data Analysis

#### 2.2.1. Codes and Themes

The codes were derived inductively from the research material. The coding tree covers the main areas, including attitudes toward vaccination, attitudes concerning COVID restrictions, the perception of the information sources, and credibility principles of the interviewees. It contains inductive and deductive codes grouped according to the subject. The main codes cover the main areas of the research, e.g., ‘Vaccine uptake’, and are supplemented by sub-codes that refer to participants’ answers. For example, ‘yes—job’ refers to the answers in which the interviewee claimed to have taken the vaccine primarily because it was required in the workplace). The number of codes and sub-codes occurrences reflects the prevalence of the views or attitudes. The coding tree is provided in the Supplementary File (Table S2).

Themes in the thematic analysis are coherent and reveal meaningful patterns [25]; however, they are not merely simple quantitative descriptions of the most frequent codes. The themes can be traced in codes’ clusters [26]. Their development was compared by Braun and Clarke to the work of sculptors rather than archaeologists, as themes are not just the names of the most frequent codes that are already there waiting to be discovered [25]. The researchers created themes on the basis of the codes and interpretations fixed in the literature and previous data.

#### 2.2.2. Analysis

TA was chosen because of its methodological clarity and accessibility for researchers with a diversified awareness of qualitative methods [26]. According to Braun and Clarke, TA concentrates on finding the patterns in data that broaden the theoretical or conceptual understanding of a research problem instead of focusing on close acquaintance with individual cases [25].

The analysis was conducted according to the six stages of Braun and Clarke’s model, as follows [25]:

1. Familiarization with the data;
2. Generating initial codes (at this stage, two research team members coded their parts of the transcripts separately with codes created during the first stage. Then, they swapped transcripts to check compliance with the codes);
3. Searching for themes;
4. Reviewing potential themes;
5. Defining and naming themes;
6. Producing the report.

In the initial phase, we distinguished 140 codes to describe interviewees’ statements about 5 subjects: COVID-19 vaccines and restrictions, the origin of the virus, sources of information, and information selection. In the next phase, this set was reduced to 65 codes that best grasped the research objectives, and approval was obtained from all of the coders. Subsequently, the codes were reduced again by merging those with similar and exchangeable meanings. It should be noted that most codes did not appear more frequently than in three interview transcripts.

We also decided to include the analysis codes with low occurrence as they reflected the opinions of interviewees with diversified attitudes toward COVID-19 vaccination: 18 not vaccinated, 4 vaccinated but strongly hesitant, and 28 vaccinated. At first, we planned to divide the interviews into two groups: vaccinated against COVID-19 and not vaccinated. However, it turned out that polarized division obscured the actual (present at the interviews) spectrum of attitudes and behaviors toward COVID-19. We decided to maintain the division into three groups during the analysis in order to grasp better the reasons why people had gotten vaccinated or not and to check if all three groups could be described by similar patterns of obtaining information and views on the origin of the new coronavirus.

We constructed themes that reflected attitudes, views, and declarations that correspond to denialist thinking. We understood ‘correspond’ to mean that they could be interpreted as such. For example, the statement ‘COVID-19 is just a flu’ would not necessarily be accompanied by other denialist statements or practices, like refusing to be vaccinated or a tendency to break restrictions imposed due to COVID-19. Thanks to that, we could trace if and what types of denialist thinking appeared among all interviewees. We created three main themes:

- Theme 1—addressing attitudes towards COVID-19 vaccines intermingled with their views on pandemic restrictions;
- Theme 2—encompassing feedback on which sources of information on COVID-19 interviewees trust and why they found some more trustworthy than the others, as well as what the explicit or implicit rules of trustworthiness are;
- Theme 3—reflecting interviewees’ views on the origins of COVID-19.

#### 2.2.3. Ethics

The study obtained the approval of the Bioethical Committee of Jagiellonian University (Decision No. 1072.6120.77.2022 from 21 April 2022). All interviewees received information about the aims and methods of the study. Before beginning the interview, they had to give informed consent. The files containing the recordings and transcriptions were named with the initials of an interviewer and consecutive ordinal numbers. The key containing the names of the interviewees and the names of the recordings was stored independently from their statements of informed consent. The recordings and transcriptions did not contain any personal data which would enable the identification of interviewees.

## 3. Results

During the analysis, we distinguished three main themes. The order of their presentation below corresponds to the importance of the subjects for interviewees and the patterns found during the analysis. Vaccination status corresponded with views on vaccination, attitudes toward the new coronavirus, and epidemic recommendations. Interviewees associated vaccines with legal regulations and restrictions that still aroused discussions and emotions. Perceptions of the sources of information correlated more with attitudes toward vaccination than other aspects of the pandemic. The differences between interviewees lay not in their preferences toward one or another specific media source but in how they assessed the veracity of information about SARS-CoV-2. The third theme, presenting views on the origins and spread of the new coronavirus, seemed to arouse the least interest among interviewees. Most of them claimed that they did not know the origin or were not interested in knowing. That could be related to the time that had elapsed since the beginning of the pandemic.

Attitudes toward vaccination against COVID-19 reflect the threefold division according to participants’ vaccination status: unvaccinated, hesitating, and vaccinated. Taking into consideration these three subgroups of interviewees, we have described subthemes that show complex attitudes toward COVID-19. Although conspiracy beliefs were the most common among unvaccinated participants, they did not all justify vaccine refusal based on conspiracy theories. A group of interviewees claimed that fear of the vaccine’s side effects was why they did not get vaccinated. For others, the main argument against vaccination was the right of free choice. It should be noted that those who took the COVID-19 vaccine also presented various views on the subject. Many arguments were considered important in decision making, from the fear of getting infected, through civil obligation, to the possibility of easier traveling.

The themes reflect the significance of the different subjects for the interviewees. It appeared that vaccines were still, after three years of the pandemic, an issue of concern for them. Their answers to the questions about vaccines also yielded the most information on the interviewees’ denialist attitudes or lack thereof. That is why that theme was described in the first position.

### 3.1. Theme 1. Attitudes toward COVID-19 Vaccination

This theme answers the research question by interpreting the interviewees’ attitudes, views, and justifications for their behavior during the COVID-19 pandemic (adherence to preventive measures, assessment of the policies, vaccine uptake) in the context of the determinants of the denialist attitude. The findings revealed that although there was a rather small group of denialists, elements of a denialist attitude could be found in all other groups. That is why this data seems sufficient to enable speaking about the spectrum of denialist attitudes that influence one’s views and behavior. In order to show the nuances in the interviews, we decided to present the theme and subthemes as presented in groups distinguished based on status and attitudes towards COVID-19 vaccination.

#### 3.1.1. The Unvaccinated Group

##### The Conspiratorial Subgroup

The most characteristic opinion expressed by this subgroup is that COVID-19 does not exist. Furthermore, vaccines should be withdrawn from healthcare services because they serve some hidden purposes not connected to health protection, e.g., for the profits of pharmaceutical companies or obtaining control over citizens. Interviewees referred to sources of information outside the mainstream media, sometimes described as ‘really independent’. Also, instead of health professionals, they referred to individuals known for promoting alternative medicine. Some representatives of this group have even openly declared that they do not believe doctors.

Below there are two examples of the statements from the ‘conspiratorial’ subgroup:

*I think that I don’t trust the messages of either political agents or vaccine companies. There were too many inaccuracies and contradictions around this topic. However, I don’t think I need such protection either.*

The next quote reveals a conspiratorial belief that the ‘truth’ about the pandemic is known only by some alternative experts, while official communiques are deceiving public opinion:

*As for COVID, I think that these vaccines should be banned altogether. I am guided by what I read on the Internet and from research that was not conducted in Poland. But as soon as the first vaccines appeared, professors immediately said what it really is. Well, it actually turned out to be true, because whoever got vaccinated now has some complications. That is not discussed in our country because everything [the media as well as governmental messages] is false.*

##### The Libertarian Subgroup

The interviewees representing the ‘libertarian’ subgroup mainly referred to freedom. They consider it unacceptable to mandate vaccination because, in their opinion, it violates civil rights:

*I believe that everyone should have the right to their own opinion, and this also repelled me a bit from the vaccine.*

Denying the existence of COVID-19 was not the only view among the ‘Libertarians’; some of them thought that the new coronavirus was not a threat to their health (‘*Every virus is a real threat. Just now, what do we mean by “threat”? Is it a threat of extinction of humanity, or just a threat of sickness?*’), although many believe contamination is dangerous for some vulnerable groups, like the elderly.

##### The Scared Subgroup

Interviewees who were scared by the possible side effects of the COVID-19 vaccine formed the biggest subgroup. They usually do not manifest other features of denialist thinking and do not believe in conspiracy theories. However, they believe that the COVID-19 vaccine was untested or tested insufficiently due to the short time of its development. The fear of side effects is so high that they decided not to get vaccinated. Similarly to the ‘Libertarians’, this group shows diversified opinions about the risk of COVID-19 infection.

Below is an example statement typical for those afraid of side effects:

*Such vaccinations should be studied for at least 5 years, if not 10. And there can be big complications later, even already now, because some side effects are already confirmed. So I think so, but not yet, and especially not for young children.*

#### 3.1.2. The Hesitating Group

##### The Companionable Subgroup

Representatives of the ‘Companionable’ subgroup were reluctant, afraid of the side effects of the COVID-19 vaccine, or they did not believe that COVID-19 was a serious threat to their health. However, ultimately, they got vaccinated only because their relatives or coworkers did it. They are still convinced that COVID-19 restrictions violated civil rights and that COVID-19 vaccination should be voluntary, but, simultaneously, most of them obeyed the restrictions. Some of them may be ‘hidden denialists’ who did not want to be perceived as such due to the pollster effect. They would not have felt comfortable revealing their denialist views to the interviewers representing the academic institution.

A typical statement in this subgroup is as follows:

*So I procrastinated, and only later, maybe under such pressure from work, or here from my family, because they were also vaccinated here. That’s why I decided to take the vaccine too.*

##### The Pragmatic Subgroup

The interviewees in the ‘Pragmatic’ subgroup have refrained from getting vaccinated but claim that they will do it if the situation forces it. Typically, they believe that COVID-19 restrictions violated civil rights, but, similarly to those from the ‘Companionable’ group, they obeyed the restrictions. The reasons for such behavior varied. Some of them were convinced that it was their civil duty. Others were forced by circumstances, e.g., they would not have been able to pursue their profession. This was the issue in this interview:

*I suspect the company, the job, might force me because later some countries introduced that unvaccinated people had to take the test, so I would probably be forced by the job or the company to take the vaccine.*

As with the ‘Companionable’ subgroup, some of them may be ‘hidden denialists’ who did not want to reveal their views to the research team. However, as opposed to the previous group, the views or decisions of their friends or colleagues were not significant to them.

#### 3.1.3. The Vaccinated Group

##### The Subgroup Showing Civic Attitude

This subgroup is characterized by the conviction that getting vaccinated and complying with restrictions was their civic duty. The interviewees in this subgroup were not afraid or were only moderately afraid of COVID-19. They could also believe that COVID-19 was only a threat to vulnerable people. They adhered to preventive behaviors, including vaccination, because they think that even if the infection can harm only a minority of the population, every citizen must do everything possible to protect them.

*At first I was a little worried, because I have vein problems and the first thing I thought was that probably it [the vaccine] can cause some kind of blockage there (…) Although my attitude towards vaccination was that it is rather a civic duty than doing it for myself.*

The interviewees did not question the existence of the new coronavirus. Sometimes, they did not perceive it as a direct danger but rather as a threat to the social or economic order. They also emphasized that the lockdowns threatened the economy and the functioning of the healthcare system, so everything possible, e.g., widespread vaccination, should be performed in order to avoid them in the future.

##### The Subgroup Afraid of the New Coronavirus

Those in this subgroup got vaccinated at the earliest opportunity because they were afraid of contracting COVID-19. Most of them did not contest pandemic restrictions and did not raise the argument that they were too severe or that they challenged civil rights. On the contrary, some interviewees saw the restrictions or recommendations as too mild.

##### The Calculating Subgroup

This group found that arguments ‘for’ prevailed over arguments ‘against’ vaccination. For example, they appreciated the possibility to travel more easily or to perform their professional duties. They did not hesitate to get vaccinated. Additionally, the ‘Calculating’ interviewees often assessed COVID-19 restrictions from the viewpoint of logic and criticized some recommendations as illogical or unfeasible. Fear of COVID-19 may have motivated them to get vaccinated and adhere to preventive behaviors, but they tried to speak about it in a more calculating manner than the interviewees in the ‘Afraid of COVID-19’ subgroup. Sometimes, they did not mention that they felt at risk at all.

Below is an example of a typical ‘calculating’ attitude. The interviewee was afraid of vaccine side effects, with the decision to get vaccinated justified by calculating the benefits and drawbacks:

*I think that everyone was a little afraid, because you know, this is a new preparation, but it is unusually… it was circulated very quickly, compared to what you usually hear about how vaccines are introduced. Although I know that it was just done in parallel different modes of testing, and not as in the normal mode. It just stretches it over time and on this basis it could be implemented faster. Well, it also turned out that side effects are very rare, but also very serious, because in some cases they could end in death. Also, you know, no matter how low the probability is, it is in your head. However, I accepted it. I decided that the benefits outweighed the risks.*

### 3.2. Theme 2. Perception of Sources of Information

Theme 2 is related to the perception of different information sources. It also describes how participants judged the credibility of information sources on COVID-19 regarding their general attitudes toward the pandemic.

Although the perception of various sources of information is one of the hallmarks of the presence or absence of a denialist attitude, some important aspects could have been omitted had it not been categorized as a separate theme. Furthermore, the way the interviewees assessed the reliability and feasibility of different sources of information appears to be an important factor strongly related to trust and competence. Three subthemes were distinguished here based on the interviewees’ comments about how they valued different sources of information. Most of the interviewees pointed to similar tools used for finding information. They used Internet browsers (‘the first page that came up in the browser’), medical practitioners, and mass media (mostly news about COVID-19 on TV and the radio). However, they saw several lines of distinction, e.g., public vs. private TV stations or Internet forums vs. institutional pages.

#### 3.2.1. The Role of Experts

Interviewees from all three groups (‘The Vaccinated’, ‘Hesitators’, and ‘The Unvaccinated’) referred to experts as the source that they trusted most. Individuals with university diplomas or with occupations relevant to the field were trusted more than intuitions or other sources (‘They invited all these different doctors and specialists onto TV programs and, so to speak, the doctors spoke wisely’.)

An interesting aspect of the analysis is how the interviewees understood who an expert was and what made them trustworthy. Most of the interviewees stressed the education and institutional affiliation of a given expert (‘Well, certainly people who have knowledge about viruses, i.e., virologists’, ‘Yes, YouTube channels, where there are people who can be verified, who are these people, so to speak’) or their employment in a governmental institution. One of the study participants said: *State institutions, or even the WHO. Some institutions that deal with epidemics or health.*

Even study participants with strong denialist attitudes claimed to trust experts. However, they referred to ‘foreign professors’ or unknown experts that were not present in the mainstream media with relevant education. Here is an example statement about such experts:

*So this is information that they have been testing for a long time and doctors confirm that it’s right. Only medics who are not paid by… To be honest, these are more like television or other independent sources that have nothing to do with state television [public TV]. When the pandemic broke out, there were a lot of professors from Italy, or some other countries, and so on…*

Below we quote a longer fragment of an interview with an unvaccinated libertarian interested in ‘psychobiology’ and ‘Germanic medicine’. ‘Germanic medicine’ (the term ‘Germanic new medicine’ is also used) can be perceived as a type of alternative medicine based on the assumption that diseases originate from unresolved conflicts experienced by individuals. In this specific context, the interviewee believed that ‘Germanic medicine’ is scientifically proven and that experts who do not acknowledge it are outdated:

*Interviewee: If I thought it was justified [to vaccinate my children], then yes, and if I thought no, then no. I would just rather form my own opinion on what will be better for us as a family.*

*Researcher: Would you discuss this with someone? For example, if your doctor encouraged you to get your child vaccinated, would you consider it?*

*Interviewee: If I felt that I got along with the doctor and that he had some knowledge about this topic, so to speak, on new discoveries. Well, I would consult him, and if I saw that he was just in such an information bubble that he only thought that we had to get vaccinated to protect ourselves against the viruses. Well, I guess I wouldn’t trust him.*

*Researcher: I see, so again it depends on this relationship and understanding that would be created or not?*

*Interviewee: And, as a result, do I think he is competent or does he just follow the current trends of science.*

Several interviewees claimed that they were in touch with experts who are their family members or friends (thus, combining the roles of a trusted friend and official expert):

*We have a doctor in our family, so I often ask and if he says “you can take it, nothing will happen to you,” or if he tells me “no,” then no. I don’t even wonder why it is so and not otherwise.*

#### 3.2.2. Common Sense

The interviewees appealed to common sense mainly when speaking about their views on COVID-19 regulations and subsequent restrictions. In relation to information reliability, for several interviewees, common sense meant not believing in extreme messages. Such an approach could have two different outcomes. On one side, it could mean a careful selection of information and the preference for official sources or sources without adverts suggesting sponsored content:

*Such credibility, the fewer colorful ads or flashing banners, the better.*

or even specific rigor in the selection of health information sources:

*If I took information from the Internet, then only from government or WHO websites. I avoided all other types of websites so as not to accidentally read the nonsense that someone had invented. Although before the first vaccine, I made sure about vaccinations, for example, for pregnant women.*

On the other side, this could also lead to difficulties in accepting pandemic restrictions or vaccination as they were considered too extreme (or improbable) to be followed (‘while some of the decisions made by the government were, in my opinion, exaggerated’).

Several interviewees, both libertarian denialists and the most disciplined citizens, recalled the prohibition on entry into the forests at the beginning of the pandemic in Poland as being illogical and unjustified. Although the prohibition lasted for only a short time, after two years, it is seen as proof that bad management or failed strategies for fighting the pandemic resulted from a lack of ‘common sense’:

*Yes, I remember the absurdity in the beginning, that we couldn’t meet more than 2 or 3 people, that we couldn’t gather, and there was no access to the forests at all. I remember a situation when we just went with friends to the forest when the ban had been lifted. Because there was a time when they first implemented it, then abolished it. And someone called the police and kindly reported that there were six of us in the woods. For me, it was really absurd to call the police, and the police had to go to great lengths to come to us, to instruct us, and they actually checked that we could be in the forest, but not the six of us, and we had to split up.*

#### 3.2.3. The Importance of Experience

Several interviewees referred to personal experience in the context of selecting information. It seems that the acceptance of the preventive measures depended on the individual experience of how they were communicated. A positive experience, e.g., feeling carefully listened to and taken care of, was particularly important when interviewees talked about trusting what health professionals told them.

*Their approach to the patient, professionalism, and the fact that I can come to them with any problem, so to speak. That I feel that doctor is taking care of me.*

Some participants appraised healthcare institutions based on their daily experience with particular doctors or nurses. It seems that a positive feeling after contact with medical staff led to higher adherence to preventive measures.

*I kind of trust doctors with whom I have been working (I don’t know if I can say so) for some time, so I trust their opinion.*

The interviewees were also inclined to refer to their existing knowledge and previous experience when they were not sure about the reliability of new information (‘*Here I am guided by my knowledge and experience*’). The acceptance of a new piece of information was determined by knowledge gained earlier. In some cases, the acceptance of information was influenced by the interviewees’ views. In the following quote, the interviewee believes that infection is an effect of stress and that it should not be treated with any prescribed medications. Thus, he evaluates the doctor based on whether recommendations are consistent with his expectations.

*I had symptoms and, of course, earlier I’d been stressed, which later manifests itself in this type of disease after resolution. So I didn’t use any medication. But I needed to take sick leave, so I had to contact a doctor. My doctor recommended that I stay at home, drink a lot water, rest, and I agreed. Somehow, I didn’t expect him to treat me like that. I thought it would be more standard here like I would have to go to the pharmacy and buy some meds. And it turned out that the doctor, I don’t know, somehow got into my... In my vision of this disease, the healing processes, and in fact it was like that and I actually recovered as I should.*

Some participants perceived not only their own but also other people’s experiences as a source of information or a way to confirm new knowledge. However, it seems there were strict, intuitive rules as to whose experience was taken into account. The information must have originated either from friends and relatives, significant others, or from people with similar traits or health problems:

*Researcher: where do you get information on health-related topics?*

*Interviewee: In most cases, in conversations with friends, when I meet them, because most often these are things that have already been used and proven [by my friends]. They can tell me how they feel after using [some meds or diet], how they use it, what the recipes are. And then I read about it on the Internet.*

### 3.3. Theme 3. The Origin of the New Coronavirus

Theme 3 revealed that participants’ views about the origin of a new coronavirus were surprisingly similar in all groups. Typically, interviewees declared a lack of interest or knowledge to speak about this issue.

#### 3.3.1. The Coronavirus Is a Natural Phenomenon

One of the most common views was that the occurrence of the new coronavirus was somehow a natural phenomenon and that it was a virus like many others. However, the reasons why it occurred and spread varied significantly, from the statements that ‘*it just happened, no one knows how*’, to more specific answers recalling ‘*bats*’ or ‘a *market in China*’. One of the interviewees claimed:

*The more of us there are in one place, the more of these viruses there will be and they will mutate and hatch into new ones. And that’s just the way it is. Well, it happened that somewhere out there this virus, so to speak, was transmitted to humans. This was certainly the case, as everyone claims, that this is the zoonotic origin of this virus. People travel around the world, they move around and that’s why it spreads easily.*

Another respondent also believed that the origin was natural. However, she asserted that she did not have the appropriate knowledge or education to speak about it: *And when it comes to the mutations themselves, the creation itself, do I know where it came from? Well, no, I’m not a specialist. No, I can’t say what happened with this specific virus. No, it wasn’t my industry, that I was involved in. I had nothing to do with coronaviruses before the pandemic, so I don’t know how they… where they originated. Well, they just happened in nature. Well, like all the others. I also don’t believe in the conspiracy theory that they were released and spread somewhere in Wuhan. Then no, no, I don’t believe in such a conspiracy theory.*

The above quotation also reflects a noticeable tendency among interviewees to avoid speculations about the origin of SARS-CoV-2.

#### 3.3.2. The Coronavirus Is Artificially Created

Some interviewees believed the COVID-19 pandemic could not be a natural phenomenon. They usually referred to its rapid spreading or the reactions of the authorities.

*It wasn’t something like someone ate a bat, but rather it was... from a laboratory. Was it something that got out on purpose or by accident, but I don’t think this COVID was born naturally, so my approach is that it probably came from the laboratory. Whether on purpose or by accident, because it also happens… But I don’t believe that it naturally somehow mutated or was naturally created by nature. (…) How fast it spread… How it works. To me, it looks like a modified flu, which, I don’t know, was intended to be used as a biological weapon, or perhaps there was simply research on this virus. Or rather, in this respect, I thought it was more like tests on this virus and that’s why it’s so contagious…*

#### 3.3.3. Spreading of the Pandemic

This subtheme refers to views of how COVID-19 spread in the population. One’s view of how the new coronavirus occurred (purposely or accidentally) in the population is not determined by one’s opinion about its origin (as a natural or an artificial phenomenon). That means that several interviewees believed that SARS-CoV-2 had developed naturally, e.g., randomly by mutations, but that the spread of the pandemic was an effect of intentional action.

There were two types of opinions about the spread of the new coronavirus in the population. Some interviewees supported the idea that it had been accidental:

*I think that laboratories are also working on various types of biological weapons, various types of vaccines, they are conducting research and, as is the case with microbes, something can always escape somewhere and if it has the conditions to spread. It will spread. And if not, no one knows anything.*

Other interviewees were more inclined to attribute the dissemination of the SARS-CoV-2 to purposeful action, but this view was less common. In this case, COVID-19 is seen as a kind of weapon or political tool, which can be interpreted as a conspiratorial belief. Below, there is a typical example of this opinion. The interviewee seems to believe in two distinct theories at the same time (the virus was spread in order to limit citizens’ freedom, and the new coronavirus is a kind of eugenical tool):

*My theory on the origin of COVID was as follows. It was a bit of a political stunt to some extent. Where, because now, it’s like China... because let’s just say it all came from China. And China likes this. They’ve subordinated their society 100 percent and have subordinated it even more. (…) And I think it was to check how people would react if they were forbidden something, because the world had been too pleasant for a long time and they wanted to see how people would react if they were forbidden something, for example, freedom. And in my opinion, this virus could have been created, for example, to attack, and for these victims to be in a community that is less necessary for productivity, so here these older people were the victims of this policy. And probably also some data on how people behave nowadays when they are forbidden to do something, who will obey, but there will be a big problem with this, and later this war happened here. And somehow it’s all a bit too strange for me.*

## 4. Discussion

Our exploratory study aimed to grasp and interpret the spectrum of denialist beliefs and attitudes among the interviewees. This required us to look for recurring arguments or explanations in interviewees’ statements while also identifying extreme statements that could indicate attitudes or views that need closer analysis. Using TA, we constructed three main themes. Most analysis was devoted to the first theme, reflecting attitudes toward COVID-19 vaccination, as its findings are the basis for the following. The typology of general attitudes helped us to analyze and interpret the phenomena in other themes. It must be stressed that, although it does not explain the full range of diversity presented in the attitudes about themes 2 and 3, the views on the new coronavirus and adherence to sanitary recommendations track with different points on the spectrum of attitudes that extend from denialism to pro-scientific, through different stages. Although the number of respondents was not enough to divide denialist attitudes into clearly defined stages and draw conclusions about the general population, it enabled us to better understand the processes that result in the prevalence of denialist attitudes.

### 4.1. Theme 1: Attitudes toward COVID-19 Vaccination

The first theme confirmed that hesitation about COVID-19 vaccination has various roots, spanning from health denialism through the fear of side effects to a libertarian outlook [27]. There is a growing corpus of scientific literature on denialism and vaccine hesitancy; however, more authors are interested in the determinants of refusal or hesitation to get vaccinated than in the motivation of those who have accepted vaccination [27,28,29,30,31,32]. Our study showed that apart from those who were simply afraid of COVID-19 infection and its consequences, several interviewees treated vaccination as their obligation. They believed that stopping the spread of the virus in the population was more important than their own views on the pandemic. In this group, there were people who were convinced that COVID-19 was a threat only to the elderly and chronically ill, not themselves, as they were young or middle-aged and healthy. For those interviewees, arguments about the common good and civil obligation were the most compelling, as they seemed consistent with their ethics [33]. Several interviewees got vaccinated for their own convenience in terms of traveling or work. Also, in this group, COVID-19 was not perceived as a big threat, but in opposition to the ‘hesitators’, they took the first possibility to receive the vaccine and mostly did not mention fearing side effects. This does not mean that they were not afraid at all, but their concerns were minor, or they decided not to mention them during the interview as they did not experience any side effects after vaccination. Also, some of the unvaccinated and ‘hesitating’ interviewees declared that the restrictions resulting from not being vaccinated could be their final reason to take the vaccine. The fear of side effects, as well as the view that the new coronavirus is a lesser threat than it is claimed to be, were more often mentioned in this group.

As shown by other authors, people who refuse to get vaccinated also present various attitudes and feelings, from denialist in a strict sense, through a mixture of different shades of denial, to first fear of side effects, and, finally, to being lost in a flood of misinformation [29]. It should be underlined that in some cases, the denialist attitude was linked to the interviewees’ libertarian values or their aversion to any extreme, with the latter also including messages from mass media or governmental recommendations. In their opinion, the scope of interference from the authorities exceeded the acceptable level.

The majority of the interviewees said that they obeyed at least some of the COVID-19-related rules, e.g., wearing a mask or maintaining distance. To some extent, preventive recommendations were even observed by participants who openly claimed that COVID-19 does not exist. Such a finding may be partially related to the pollster effect. The interviewees felt uncomfortable confessing to a research team from the Faculty of Health Sciences that they had not followed epidemiological rules. Some of the participants were generally in favor of rules that seemed transparent, logical, and justified. Please see the quotation above about the restrictions imposed on entering forests during the first phase of the pandemic in Poland. There were also voices that claimed that obeying pandemic rules in public transportation or during some professional activities was nearly impossible.

Quite a few interviewees admitted that they obeyed rules only when they had to, e.g., because of the imposed fines or if the number of clients in a shop was controlled by the staff. It also seems that even individuals with hard denialist attitudes did not break the rules if there was not an ‘atmosphere’ for that. As such, if most of the people around them accepted the rules and adhered to preventive behaviors, e.g., wore masks indoors, denialists toed the line. Apparently, denialist attitudes were not able to overcome conformism.

Attitudes toward the recommended preventive measures and related regulations generally depended on individuals’ views about the COVID-19 pandemic. We could observe the whole spectrum of attitudes, from total refusal of the introduced restrictions among interviewees who questioned the existence of the pandemic to dissatisfaction with their radicalness among those who perceived the pandemic as an imminent danger.

Complaints about problems with governmental communication to citizens during the pandemic were common in the interviews. They were expressed by interviewees independently of their convictions about the existence and risks associated with COVID-19 infection. Apart from insufficient communication, participants were also unsatisfied with explanations for quickly changing regulations. Our findings seem to contradict the results of early public opinion polls conducted in Poland that showed that nearly half of Poles had positively assessed the government’s actions [34]. Obviously, in the early phase of the pandemic, people were more inclined to follow governmental instructions than two years later, when the pandemic was over.

The crisis regarding trust in governmental and public health organizations as a determinant of COVID-19 vaccine hesitancy has been reported on previously by authors of synthetic reports [15,16]. According to Scandurra et al., lower trust in governmental organizations increases future anxiety and one’s level of fatigue, leading finally to a decreased level of protective behavior [35]. People with higher levels of trust were more compliant with public health regulations during the second wave of the COVID-19 pandemic [35]. A large study performed in 23 countries titled the ‘PsyCorona Survey’ also revealed that trust in government was significantly associated with adopting preventive behaviors [36]. The perception that the government was well organized and disseminating clear messages and knowledge on COVID-19, as well as the perception that governmental actions were fair, were the determinants of trust in government. Finally, according to Liu and Huang, the quality of the relationship between local government and the public could predict the acceptance of COVID-19 vaccination [37].

The attitudes associated with health denialism, e.g., questioning the existence or severity of the pandemic or refusing to get vaccinated, were frequently accompanied by a lack of trust in modern democratic institutions (the media, experts, and the government). This observation has been shared by other authors who have studied the adherence to preventive measures during the pandemic [31].

In our study, this distrust was expressed with varying intensity and targets. Some of the interviewees were skeptical about the messages received from the mass media, the government, experts, or combinations of these institutions. Denialist attitudes were more often associated with the mistrust of all three entities. Those who felt lost amidst the information noise tended to lose trust in mass media.

The interviews clearly showed that denialism was one of the most important reasons why people did not get vaccinated or obey the pandemic rules. However, it remained ‘in the shadows’ in many interviews, and the participants did not overtly manifest all of the typical attitudes defined by the Hoofnagles [1]. On the other hand, some elements of denialist attitudes could be identified in all groups of interviewees distinguished in our analysis. Clearly, we observed some propensity toward conspiracy beliefs, especially associated with vaccination. We had also previously marked a high prevalence of conspiracy beliefs regarding COVID-19 in Polish society [10,32].

In our current study, independently of the interviewee’s COVID-19 vaccination status, there were those who were scared of side effects. Furthermore, the claim that the COVID-19 vaccines had not been tested or had been tested insufficiently was very common in the interviews. Aside from conspiracy theories, a feeling of uncertainty and a readiness to question the intentions of the authorities were common, mainly due to contradictory information about the new coronavirus in mass and social media, as well as rapid, non-transparent changes in official regulations.

Doubts about the safety and effectiveness of COVID-19 vaccines were an important factor responsible for vaccine hesitancy, especially under overwhelming misinformation [16]. The attitudes toward the COVID-19 vaccination underwent considerable changes in various countries. Saleh et al. observed that in the first year of the pandemic, the sentiment about COVID-19 vaccination assessed in English language tweets changed positively, moving from a position of fear being more prevalent to rather one of trust [38]. However, later studies showed considerable shifts in hesitancy and acceptance of COVID-19 vaccination hesitancy. Chen et al. reported that the rate of vaccine hesitancy fluctuated significantly between April 2021 and December 2022 in China [39]. It dropped from 30% in April 2021 to 13.7% in November but increased to 30.4% in December 2022. The study, conducted in 23 countries, revealed that between June 2021 and June 2022, the acceptance of the COVID-19 vaccine increased in 17 and decreased in 8 countries [40]. In Poland, this increase reached 8%; however, it should be underlined that the level of hesitancy in Polish society, measured in June 2022, was still one of the highest (nearly 36%). Apparently, worries incited during the development of the COVID-19 vaccine and reports suggesting limited effectiveness or serious side effects of new vaccines after their wide distribution have persisted in Polish society.

Motta and Stecula also found that messaging regarding COVID-19 side-effects based on the national reporting system was characterized differently depending on the political perspective of the given media outlet. As a result, some news channels promoted the emergence of significantly more negative sentiments about the COVID-19 vaccine, propelling the increase in vaccine hesitancy [41]. It also seems that the perceived risk of side effects following COVID-19 vaccination may lead to low acceptance of booster doses [40]. The study performed by Razzaghi et al. in April 2023 among pregnant women in the USA revealed that only 27.3% of them received a COVID-19 bivalent booster dose [42].

### 4.2. Theme 2: Perception of the Sources of Information

Here, again, most of the interviewees defy binary divisions. The interviewees demonstrating some denialist views trusted experts and specialists with university degrees to nearly the same degree as other participants. More detailed analysis revealed that the perceived reliability of an expert depended on (1) the expert’s affiliation, (2) the way of communication, and (3) previous contacts with the health professionals. Not surprisingly, hard denialists preferred specialists who spoke in niche media and spoke out against the officials. As for the way of communication, the experts with more balanced opinions and those whose perspectives were consistent with the interviewee’s knowledge and worldview were preferred. The interviewees who assessed their previous contacts with health professionals as satisfying (e.g., they received helpful advice) were more prone to trust the opinions expressed by health professionals regarding current issues.

Moreover, analysis showed that none of the interviewees had claimed not to believe in experts and scientific knowledge, even those whose views could be interpreted as denialist. Our interviews showed that trust in scientific knowledge was not an issue of debate but was instead variously conditioned. Factors, such as trust in institutions and the authorities, individual and cultural values and experiences, and being competent to distinguish evidence-based knowledge from opinions have also been described by other authors [43,44]. Several interviewees seemed to rely on their knowledge and experience on COVID-19 more than any other source of information. This can also be interpreted as an effect of the mistrust in institutions that led to hesitation in vaccine uptake [45].

### 4.3. Theme 3: The Origins of the New Coronavirus

When asked about their opinions on the origin of the new coronavirus, most of the interviewees answered that they did not know, did not have the ability or knowledge to respond, or were simply just not interested in it. Such responses can be interpreted as a type of escape option often used in surveys, e.g., ‘Don’t know’ [46].

The remaining participants provided various explanations, sometimes pointing to conspiracy theories, and sometimes combining them. For example, the same interviewee could claim that the new coronavirus had been created in a laboratory and was spread to the population as the result of an accident.

An in-depth analysis of the interviewees’ feedback on the origin of the COVID-19 pandemic revealed interesting aspects. Many interviewees were not able to say when the pandemic had begun, mainly because it was no longer a subject of their everyday discussions or of the news distributed in the mass media. The pandemic had simply stopped being an important issue in public opinion.

We can observe a complex interplay between mistrust and the infodemic. This term was introduced early during the COVID-19 pandemic to indicate the overwhelming quantity of disinformation accompanying the epidemiological phenomenon [47]. The infodemic that occurred during the pandemic resulted in mistrust in the media as well as difficulties for many interviewees in forming their own opinions about the phenomenon if they had had no personal experience of it. The mistrust in the mass media was, to some extent, responsible for the evasive responses about the origin of the pandemic. Interviewees preferred to select the equivalent of the response ‘don’t know’ as they were not convinced by or felt they lacked official information about COVID-19.

Feeling uncomfortable with the news distributed by mass media due to shock tactics to gain audience interest or how quickly contradictory information emerged, some of the interviewees looked for information on Internet forums, which was provided by individuals deemed experts due to their education or experience. A previous study showed that people are vulnerable to disinformation if it looks like a statement from a common person [48]. Another outlook is that there is a connection between bias against extreme messages, appealing to common sense, and ‘escape responses’, which fit into the image of a ‘normal’, measured person. Being measured is culturally highly appreciated [49]. Wanting to present a measured appearance could have influenced the interviewees, as at least some of them may have wanted to make such an impression on the interviewer. Summing up, it turned out that, outside of a few cases, views on the origin of the new coronavirus were rather indistinct.

### 4.4. Limitations of the Study

The interviews were conducted after almost 2.5 years of the pandemic, at a time when the number of infections and deaths was much lower than in 2020 and 2021, and officials stated that the threat had been controlled. Moreover, other events, such as the escalation of the war in Ukraine, an influx of refugees, and growing inflation, commanded the interest of public opinion. Both mass media and governmental institutions stopped pro-vaccine campaigns. All of this might have affected the willingness of the interviewees to speak about their attitudes toward vaccination and the new coronavirus. The passage of time may have caused them to simply forget many things that they might have considered important at the beginning of the pandemic.

The semi-structured interview guide did not include whether an interviewee had contracted COVID-19. Such information was only registered if the interviewee provided it spontaneously. We tried to avoid questions that could be too intrusive for participants.

The study utilized qualitative methods, and its results cannot be generalized. Although the research team made every possible effort to diversify the study sample, the specificity of the method does not ensure that it reflects the structure of the Polish population, and its size makes it impossible to draw general conclusions. Furthermore, we faced 11 refusals to participate in the interview; this is a relatively large group, considering the final number of conducted interviews. It also seems that those who did not agree to join the study could present more extreme denialistic views. We believe that this could be a limitation of the study. However, we were determined to assess the prevalence of denialistic views in representatives of the general population, and recruitment was not selective regarding such attitudes.

It should also be noted that the conclusions refer to subjective opinions, although all possible steps were taken to see that the data was saturated. In different times, other countries, or settings, the data would differ, and analysis could give different results. The analysis is based on interpretations made by the research team. Although at every stage, we made all possible efforts to justify the interpretations included in the analysis by referencing the available literature, this analysis is not free of subjective views. This was, however, inevitable when one considers the aim of exploring the interviewees’ beliefs and arguments.

## 5. Conclusions

We have conducted IDIs during a late phase of the COVID-19 pandemic in Poland to understand how health denialism manifested in interviewees’ attitudes towards the origin of the new coronavirus and COVID-19 vaccination.

Contrary to observations from earlier phases of the pandemic, the study participants did not report definite opinions about the origin of COVID-19. Most commonly, they provided evasive responses. Only some of them repeated conspiracy theories which had been extremely popular when the pandemic erupted.

As for the adherence to preventive measures, we could observe an interplay of diversified motivations and attitudes. The interviewees tended to have differing opinions about specific rules recommended during the pandemic. Furthermore, even individuals with strong denialist views admitted that they had submitted to dominating attitudes in specific situations, e.g., they wore masks if the majority of people in the room were doing so.

It should be emphasized that health denialism, both related to the perception of the COVID-19 pandemic and the vaccine against SARS-CoV-2, was one of the main determinants of vaccine hesitancy and the refusal to get vaccinated.

## Figures and Tables

**Table 1:** Characteristics of the study sample.

Variable	Categories of Variable	%	*n*
Age	18–40	58.0	29
	41–65	38.0	19
	>65	4.0	2
Sex	Male	52.0	26
	Female	48.0	24
Place of residence	Rural	32.0	16
	Urban below 50,000	16.0	8
	Urban from 50,000 to below 100,000	8.0	4
	Urban from 100,000	44.0	22
Education	Secondary or post-secondary non-tertiary	44.0	22
	University degree	56.0	28
Vocational status	Employee or self-employed	82.0	41
	Retired or on disability pension	12.0	6
	University or school student	4.0	2
	Unemployed	2.0	1
Marital status	Single	24.0	12
	Widowed/divorced/in separation	4.0	2
	Married	50.0	25
	In partnership	22.0	11
Average time spent on the Internet per day	<30 min.	4.0	2
	30–60 min.	24.0	12
	>60–120 min.	39.0	15
	>120 min.	42.0	21
COVID-19 vaccination	Yes	84.0	42
	No	16.0	8

## Data Availability

The dataset generated and analyzed during the current study are available in the ZENODO repository [50].

## Supplementary Materials

The following supporting information can be downloaded at: https://www.mdpi.com/article/10.3390/vaccines11121822/s1, Table S1: Semi-structured interview guide; Table S2: Coding tree; Table S3: COREQ Form.
