# Supplementary material for: Manifestation of Health Denialism in Attitudes toward COVID-19 Vaccination: A Qualitative Study
Source: Vaccines (Basel). 2023 Dec 6;11(12):1822. doi: 10.3390/vaccines11121822 (PMC10747861; doi:10.3390/vaccines11121822)
Supplement: Supplementary file 1 [file vaccines-11-01822-s001.zip › vaccines-2713979-supplementary.pdf]

Supplementary materials

Table S1. The interview guide

Topic	Questions
Adherence to sanitary recommendations	Over the last 2 years, many regulations, orders and prohibitions related to the COVID-19 pandemic have been introduced. Have you complied with them<br>How has it changed over time?<br>What guided you when deciding whether or not to follow a specific recommendation?
Perception of threat	Do you think that the threat related to COVID is real?<br>Do you personally feel fear or concern about the possibility of contracting COVID-19?
The origin of COVID-19	Where do you think the new coronavirus came from (how did the pandemic start)?<br>What other theories/views about the beginnings of the pandemic do you know?<br>Which of them do you think are true?<br>What views on the COVID-19 pandemic are popular among your friends/family?
Sources of information	Where does your knowledge about COVID-19 come from?<br>Are you specifically looking for information on this topic?<br>Do your friends/family give you advice on how to act during the pandemic on a daily basis?<br>If you were to indicate the three most reliable sources of information on the coronavirus, those that you have used or would do so, what would they be?
Views on vaccines against COVID-19	What does you think about COVID-19 vaccines?<br>Are you afraid of vaccines?<br>Imagine what could convince you not/ to get vaccinated<br>Have you decided to get vaccinated?

Table S2. The coding tree

Code	Number of occurrences
Coronavirus origin: doesn't know – no official statement	2
Coronavirus origin: natural	3
Coronavirus origin: artificial	2
Coronavirus origin: lack of interest	11
Coronavirus origin: does not know/can't evaluate info	14
Coronavirus: fried purposely	2
COVID-19 danger: no – it is like a flu	4
COVID-19 danger: no - an artificially created threat	4
COVID-19 danger: no – is not afraid at all	2
COVID-19 danger: no, but fear of hospital	1
COVID-19 danger: yes – not very big threat	3
COVID-19 danger: yes – for interviewee	3
COVID-19 danger: yes – for other people	10
COVID-19 information - information noise	5
COVID-19 information – hiding the truth	1
Information: selection – friends-experts (having university degrees or working in the field of interest)	10
Information: selection – independent/alternative experts	5
Information: selection – legitimized experts	19
Information: selection – independent/alternative experts	5
Information: selection – friends-experts (having university degrees or working in the field of interest)	10
Information: selection on the Internet – compatibility with prior knowledge	6
Information: selection on the Internet – opinions	2
Information: selection on the Internet – repetition in various sources	7
Information: selection on the Internet – types of web pages linked to	2
Information: selection on the Internet – adverts	4
Information: selection on the Internet – known pages	2
Information: sources - TV	8
Information: sources - books	3
Information: sources – other (radio+newspaper+"different")	3
Information: sources – Internet forums	3
Information: sources – Internet browser	20
Information: sources – web pages of institutions (Ministry of Health, World Health Organisation etc.)	8
Information: sources – various (crossbreeding, crosschecking)	14
Pandemic: bad organisation	8
Pandemic: social problem	2
Pandemic: political issue	3

Pandemic: media/news isuue	5
Pandemic: fear management	4
Pandemic restrictions&injunctions: no – illogical	1
Pandemic restrictions&injunctions: selectively – doesn’t cope well being lock down	1
Pandemic restrictions&injunctions: selectively – common sense	11
Pandemic restrictions&injunctions: yes – friends’ experience	1
Pandemic restrictions&injunctions: yes - penalties	4
Pandemic restrictions&injunctions: yes – social/citizen obligation	7
Pandemic restrictions&injunctions: yes – subordination	5
Pandemic restrictions&injunctions: yes – passively	2
Pandemic restrictions&injunctions: yes – legalism (obeying the law is important value)	5
Pandemic restrictions&injunctions: yes – precaution	12
Pandemic restrictions&injunctions: yes – fear	3
Pandemic restrictions&injunctions: adherence changing over time	10
Pandemic restrictions&injunctions: illogical	4
Pandemic restrictions&injunctions: restriction of freedom	4
Pandemic restrictions&injunctions: some good practices/still useful	4
Vaccine – for vulnerable groups	2
Vaccine – product	1
Vaccine - voluntary	5
Vaccine danger: fear of side effects	3
Vaccine danger: no	6
Vaccine uptake : conditional in the future – if needed in the job	2
Vaccine uptake : conditional in the future – if pandemic lasted	2
Vaccine uptake : conditional in the future – if relatives get sick	1
Vaccine uptake : no – do not need it	3
Vaccine uptake : no – influence on the organism	4
Vaccine uptake : no – never get sick	4
Vaccine uptake : no – not tested enough	9
Vaccine uptake : no – should be banned	2
Vaccine uptake yes – because others took	3
Vaccine uptake yes – civic obligation	2
Vaccine uptake yes - job	6
Vaccine uptake yes - trip	3
Vaccine uptake yes – protection	6
Value: choice	4
Value: openness (on opinions)	3

Table S3. The COREQ form

Topic	Item no	Guide Questions/Description	Reported on page no
Domain 1: Research team and reflexivity	1. Interviewer/facilitator	Which author/s conducted the interview or focus group?	5
	2. Credentials	What were the researcher's credentials?	5
	3. Occupation	What was their occupation at the time of the study?	5
	4. Gender	Was the researcher male or female?	1
	5. Experience and training	What experience or training did the researcher have?	5
	6. Relationship established	Was a relationship established prior to study commencement?	5
	7. Participant knowledge of the interviewer	What did the participants know about the researcher? e.g. personal goals, reasons for doing the research	7
	8. Interviewer characteristics	What characteristics were reported about the inter viewer/facilitator? e.g. Bias, assumptions, reasons and interests in the research topic	5
Domain 2: Study design	9. Methodological orientation and Theory	What methodological orientation was stated to underpin the study? e.g. grounded theory, discourse analysis, ethnography, phenomenology, content analysis	3-4
	10. Sampling	How were participants selected? e.g. purposive, convenience, consecutive, snowball	4
	11. Method of approach	How were participants approached? e.g. face-to-face, telephone, mail, email	4
	12. Sample size	How many participants were in the study?	4
	13 Non-participation	How many people refused to participate or dropped out? Reasons?	5
	14. Setting of data collection	Where was the data collected? e.g. home, clinic, workplace	4
	15. Presence of non-participants	Was anyone else present besides the participants and researchers?	4
	16. Description of sample	What are the important characteristics of the sample? e.g. demographic data, date	4
	17. Interview guide	Were questions, prompts, guides provided by the authors? Was it pilot tested?	4
	18. Repeat interviews	Were repeat inter views carried out? If yes, how many?	4
	19. Audio/visual recording	Did the research use audio or visual recording to collect the data?	5
	20. Field notes	Were field notes made during and/or after the interview or focus group?	5
	21. Duration	What was the duration of the inter views or focus group?	5
	22. Data saturation	Was data saturation discussed?	5
	23. Transcripts returned	Were transcripts returned to participants for comment and/or corrrection	5
	24. Number of coders	How many data coders coded the data?	5

Domain 3: analysis and findings	25. Description of the coding tree	Did authors provide a description of the coding tree?	5-6
	26. Derivation of themes	Were themes identified in advance or derived from the data?	6
	27. Software	Derivation of themes	6
	28. Participant checking	Did participants provide feedback on the findings?	5
	29. Quotations presented	Were participant quotations presented to illustrate the themes/findings?	6-15
	30. Data and findings consistent	Was there consistency between the data presented and the findings?	6-15
	31. Clarity of major themes	Were major themes clearly presented in the findings?	6-15
	32. Clarity of minor themes	Is there a description of diverse cases or discussion of minor themes?	6-15, 16-18
